# Crystal structure of (3-carb­oxy­prop­yl)tri­phenyl­phospho­nium hexa­fluorido­phosphate

**DOI:** 10.1107/S160053681402323X

**Published:** 2014-10-24

**Authors:** Patrick C. Hillesheim, Kent A. Scipione, Sean L. Stokes

**Affiliations:** a Mississippi State University, Department of Chemistry, 1115 Hand Lab, Box 9573, Mississippi State, MS 39762, USA

**Keywords:** crystal structure, phospho­nium salt, hydrogen bonding

## Abstract

In the title mol­ecular salt, C_22_H_22_O_2_P^+^·PF_6_^−^, the side chain of the cation adopts an *anti–gauche* conformation [P—C—C—C and C—C—C—C torsion angles = −179.11 (10) and −77.18 (16)°, respectively]. In the crystal, the cations are linked into carb­oxy­lic acid inversion dimers by pairs of O—H⋯O hydrogen bonds. Weak C—H⋯F and C—H⋯(F,F) hydrogen bonds connect the components into a three-dimensional network, but there are no aromatic π–π stacking inter­actions.

## Related literature   

For structures of related compounds, see: Li & Mak (1996 ▶); Wu *et al.* (2007 ▶). For compounds containing related metallated structures, see: Li & Mak (1997 ▶); Sabounchei *et al.* (2011 ▶). For the use of phospho­nium compounds as Wittig reagents, see: Hoffman (2001 ▶), as biocodal agents, see: Kanazawa *et al.* (1993 ▶) and as phase transfer agents, see: Starks (1971 ▶).
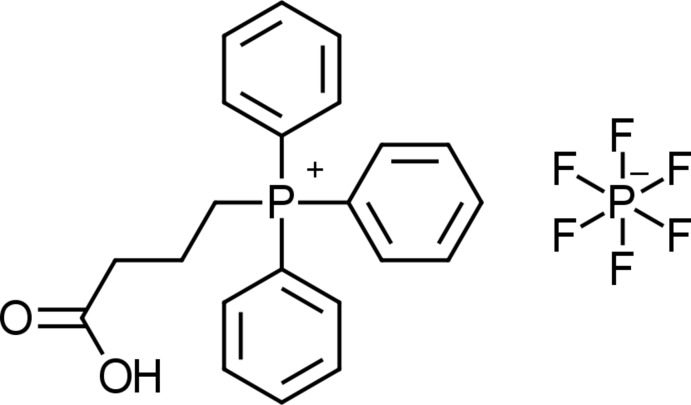


## Experimental   

### Crystal data  

C_22_H_22_O_2_P^+^·PF_6_^−^
*M* *_r_* = 494.33
Triclinic, 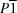

*a* = 9.3307 (1) Å
*b* = 10.6773 (2) Å
*c* = 12.8129 (2) Å
α = 72.460 (1)°
β = 82.307 (1)°
γ = 65.495 (1)°
*V* = 1107.46 (3) Å^3^
*Z* = 2
Mo *K*α radiation
μ = 0.26 mm^−1^
*T* = 100 K
0.29 × 0.16 × 0.07 mm

### Data collection  

Bruker APEXII CCD diffractometer
Absorption correction: multi-scan (*SADABS*; Bruker, 2014 ▶) *T*_min_ = 0.865, *T*_max_ = 0.947
37843 measured reflections
5269 independent reflections
4426 reflections with *I* > 2σ(*I*)
*R*_int_ = 0.035

### Refinement  

*R*[*F* ^2^ > 2σ(*F* ^2^)] = 0.034
*wR*(*F* ^2^) = 0.087
*S* = 1.06
5269 reflections
290 parameters
H-atom parameters constrained
Δρ_max_ = 0.42 e Å^−3^
Δρ_min_ = −0.36 e Å^−3^

### 

Data collection: *APEX2* (Bruker, 2014 ▶); cell refinement: *SAINT* (Bruker, 2013 ▶); data reduction: *SAINT*; program(s) used to solve structure: *SHELXT* (Sheldrick, 2008 ▶); program(s) used to refine structure: *SHELXL2014* (Sheldrick, 2008 ▶); molecular graphics: *OLEX2* (Dolomanov *et al.*, 2009 ▶); software used to prepare material for publication: *OLEX2*.

## Supplementary Material

Crystal structure: contains datablock(s) I. DOI: 10.1107/S160053681402323X/hb7304sup1.cif

Structure factors: contains datablock(s) I. DOI: 10.1107/S160053681402323X/hb7304Isup2.hkl

Supporting information file. DOI: 10.1107/S160053681402323X/hb7304Isup3.mol

**Fig. 1.**
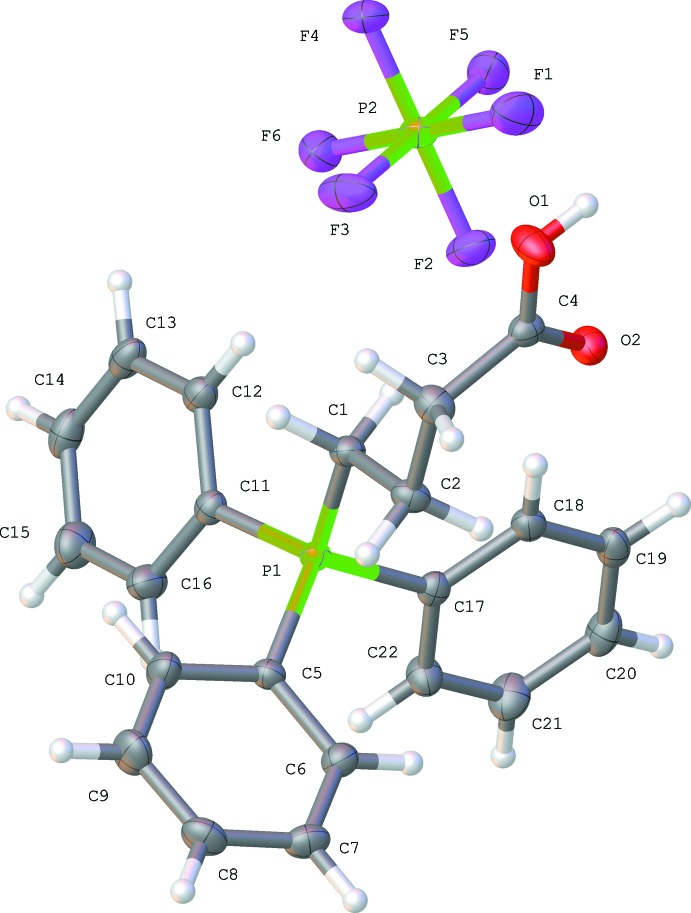
Crystal structure and labeling scheme of compound (1). 50% probablility ellipsoids. Phosphorous is in green, oxygen in red, fluorine in purple, and carbon in grey.

. DOI: 10.1107/S160053681402323X/hb7304fig1.tif
Crystal structure and labeling scheme of compound (1). 50% probablility ellipsoids. Phospho­rous is in green, oxygen in red, fluorine in purple, and carbon in grey.

CCDC reference: 1030392

Additional supporting information:  crystallographic information; 3D view; checkCIF report

## Figures and Tables

**Table 1:** Hydrogen-bond geometry (, )

*D*H*A*	*D*H	H*A*	*D* *A*	*D*H*A*
O1H1O2^i^	0.84	1.80	2.6285(15)	171
C1H1*A*F2	0.99	2.48	3.455(2)	168
C1H1*A*F3	0.99	2.50	3.1656(19)	124
C22H22F4^ii^	0.95	2.51	3.3924(18)	155

Symmetry codes: (i) 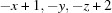; (ii) 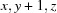.

## supplementary crystallographic information

### S1. Synthesis and crystallization

A 1.0g (2.3mmol) sample of 3-carb­oxy­propyl­tri­phenyl­phospho­nium chloride and 0.4g (2.4mmol) of sodium hexa­fluoro­phosphate were dissolved in 40mL of water. A white precipitate immediately formed and the slurry was stirred for 1 hour. The mixture was filtered, the solid was washed with water (3 x 25mL), and dried under high vacuum to yield a white solid. Yield: 0.65g (80.8%). Single crystals suitable for X-ray diffraction were grown from slow evaporation of di­chloro­methane. ^1^H NMR (CHLOROFORM-*d* ,300MHz): δ = 7.90–8.02 (*m*, 9H), 7.77–7.89 (*m*, 6H), 3.54–3.72 (*m*, 2H), 2.66 (*t*, *J* = 6.6 Hz, 2H), 2.04–2.07 p.p.m. (*m*, 2H). ^13^C NMR (CHLOROFORM-*d* ,75MHz): δ = 174.0, 136.4, 134.9, 131.6, 120.3, 119.1, 34.2, 34.0, 22.0, 19.2 p.p.m. HRMS (ESI–TOF) *m*/*z*: [*M*^+^] Calcd for C_22_H_22_O_2_P^+^ 349.381; found 349.1355. [*M*^-^] Calcd for PF_6_ 144.965; found 144.9632.

### S2. Refinement

Carbon-bound H-atoms were placed in calculated positions (C—H = 0.95 to 0.99 Å, O—H = 0.84 Å) and were included in the refinement in the riding model approximation with *U*_iso_(H) = 1.2*U*_eq_(C) or 1.5*U*_eq_(O).

### S3. Comment

Organic phosphonium cations have been used as phase transfer catalysts (Starks, 1971), biocidal agents (Kanazawa *et al.* 1993), and as reagents for Wittig reactions (Hoffman, 2001). There are few examples in the crystallographic literature, however, of triphenylphosphonium cations bearing a carboxylic acid functional group.

In the title compound, 3-carboxypropyltriphenylphosphonium hexafluorophosphate (Fig. 1), crystallizes in a triclinic unit cell with a single cation-anion pair in the asymmetric unit. The dominant intermolecular interactions is hydrogen bonding from the carboxylic acid moiety on the cation (Table 1). The alkyl chain attached to the phosphorous deviates from the expected staggered conformation, showing a rotation at the C1—C2 carbons. This twist in the carbons is likely the cause of the unusual torsion angles observed in the three phenyl rings (Table 2). The phenyl ring that is located under the C2 hydrogens is nearly perpendicular when compared to the other two rings. It is suspected that this perpendicular arrangement of the phenyl ring is assumed to minimize potential steric interactions with the bent portion of the alkyl chain. Interestingly, there are no observed π–π interactions from any of the phenyl rings and there are no weak C—H···F interactions.

### Crystal data

**Table:**

C_22_H_22_O_2_P^+^·PF_6_^−^	*Z* = 2
*M**_r_* = 494.33	*F*(000) = 508
Triclinic, *P*1	*D*_x_ = 1.482 Mg m^−^^3^
*a* = 9.3307 (1) Å	Mo *K*α radiation, λ = 0.71073 Å
*b* = 10.6773 (2) Å	Cell parameters from 9960 reflections
*c* = 12.8129 (2) Å	θ = 2.4–27.8°
α = 72.460 (1)°	µ = 0.26 mm^−^^1^
β = 82.307 (1)°	*T* = 100 K
γ = 65.495 (1)°	Block, colourless
*V* = 1107.46 (3) Å^3^	0.29 × 0.16 × 0.07 mm

### Data collection

**Table:**

Bruker APEXII CCD diffractometer	5269 independent reflections
Radiation source: fine-focus sealed tube	4426 reflections with *I* > 2σ(*I*)
Graphite monochromator	*R*_int_ = 0.035
Detector resolution: 7.9 pixels mm^-1^	θ_max_ = 27.9°, θ_min_ = 1.7°
ω and φ scans	*h* = −12→11
Absorption correction: multi-scan (*SADABS*; Bruker, 2014)	*k* = −14→14
*T*_min_ = 0.865, *T*_max_ = 0.947	*l* = −16→16
37843 measured reflections	

### Refinement

**Table:**

Refinement on *F*^2^	Primary atom site location: structure-invariant direct methods
Least-squares matrix: full	Secondary atom site location: difference Fourier map
*R*[*F*^2^ > 2σ(*F*^2^)] = 0.034	Hydrogen site location: inferred from neighbouring sites
*wR*(*F*^2^) = 0.087	H-atom parameters constrained
*S* = 1.06	*w* = 1/[σ^2^(*F*_o_^2^) + (0.0364*P*)^2^ + 0.4848*P*] where *P* = (*F*_o_^2^ + 2*F*_c_^2^)/3
5269 reflections	(Δ/σ)_max_ = 0.001
290 parameters	Δρ_max_ = 0.42 e Å^−^^3^
0 restraints	Δρ_min_ = −0.36 e Å^−^^3^

### Special details

**Table:**

Experimental. Absorption correction: SADABS-2014/2 (Bruker, 2014) was used for absorption correction. wR2(int) was 0.0583 before and 0.0488 after correction. The Ratio of minimum to maximum transmission is 0.9133. The λ/2 correction factor is 0.00150.
Geometry. All e.s.d.'s (except the e.s.d. in the dihedral angle between two l.s. planes) are estimated using the full covariance matrix. The cell e.s.d.'s are taken into account individually in the estimation of e.s.d.'s in distances, angles and torsion angles; correlations between e.s.d.'s in cell parameters are only used when they are defined by crystal symmetry. An approximate (isotropic) treatment of cell e.s.d.'s is used for estimating e.s.d.'s involving l.s. planes.

### Fractional atomic coordinates and isotropic or equivalent isotropic displacement parameters (Å^2^)

**Table:**

	*x*	*y*	*z*	*U*_iso_*/*U*_eq_	
P1	0.42475 (4)	0.61911 (4)	0.72374 (3)	0.01582 (9)	
P2	0.21110 (5)	0.22893 (4)	0.72342 (3)	0.02490 (10)	
F2	0.18704 (11)	0.33829 (11)	0.79338 (8)	0.0344 (2)	
F6	0.13566 (12)	0.36150 (10)	0.61884 (8)	0.0352 (2)	
F4	0.23378 (12)	0.12048 (10)	0.65303 (8)	0.0342 (2)	
F5	0.03862 (12)	0.23173 (12)	0.76017 (8)	0.0387 (3)	
F3	0.38172 (12)	0.22834 (11)	0.68553 (9)	0.0406 (3)	
O2	0.48983 (12)	0.17127 (10)	0.96794 (8)	0.0235 (2)	
F1	0.28391 (14)	0.09741 (11)	0.82789 (9)	0.0453 (3)	
O1	0.66794 (13)	−0.01961 (11)	0.91753 (10)	0.0295 (3)	
H1	0.6104	−0.0598	0.9557	0.044*	
C5	0.58121 (16)	0.67621 (14)	0.71818 (11)	0.0172 (3)	
C18	0.23325 (17)	0.57402 (16)	0.90634 (11)	0.0208 (3)	
H18	0.2785	0.4755	0.9084	0.025*	
C11	0.32167 (16)	0.70037 (15)	0.59658 (11)	0.0184 (3)	
C17	0.28593 (16)	0.67064 (15)	0.82977 (11)	0.0182 (3)	
C4	0.60945 (17)	0.11522 (15)	0.91839 (11)	0.0200 (3)	
C1	0.51033 (16)	0.42771 (14)	0.74813 (12)	0.0188 (3)	
H1A	0.4262	0.3910	0.7713	0.023*	
H1B	0.5569	0.4039	0.6789	0.023*	
C12	0.25839 (16)	0.62504 (16)	0.55808 (12)	0.0217 (3)	
H12	0.2804	0.5273	0.5944	0.026*	
C2	0.63811 (16)	0.35274 (15)	0.83609 (12)	0.0207 (3)	
H2A	0.7222	0.3896	0.8137	0.025*	
H2B	0.5918	0.3743	0.9060	0.025*	
C6	0.62009 (17)	0.69945 (15)	0.80985 (12)	0.0213 (3)	
H6	0.5526	0.7019	0.8723	0.026*	
C19	0.11368 (17)	0.62379 (17)	0.97956 (12)	0.0243 (3)	
H19	0.0781	0.5585	1.0325	0.029*	
C22	0.22021 (17)	0.81526 (15)	0.82747 (12)	0.0239 (3)	
H22	0.2574	0.8806	0.7762	0.029*	
C13	0.16332 (17)	0.69379 (18)	0.46650 (12)	0.0255 (3)	
H13	0.1208	0.6429	0.4394	0.031*	
C3	0.70880 (17)	0.19153 (15)	0.85265 (13)	0.0240 (3)	
H3A	0.7292	0.1726	0.7799	0.029*	
H3B	0.8116	0.1506	0.8893	0.029*	
C14	0.13030 (18)	0.83680 (18)	0.41448 (12)	0.0276 (3)	
H14	0.0626	0.8843	0.3530	0.033*	
C16	0.29145 (19)	0.84353 (16)	0.54243 (13)	0.0262 (3)	
H16	0.3363	0.8942	0.5677	0.031*	
C7	0.75827 (17)	0.71902 (16)	0.80929 (13)	0.0253 (3)	
H7	0.7852	0.7355	0.8714	0.030*	
C8	0.85698 (18)	0.71457 (17)	0.71847 (14)	0.0275 (3)	
H8	0.9528	0.7254	0.7193	0.033*	
C10	0.67893 (18)	0.67535 (17)	0.62542 (12)	0.0251 (3)	
H10	0.6511	0.6618	0.5622	0.030*	
C21	0.10050 (19)	0.86258 (17)	0.90047 (13)	0.0296 (3)	
H21	0.0552	0.9609	0.8990	0.035*	
C20	0.04614 (17)	0.76728 (18)	0.97598 (12)	0.0271 (3)	
H20	−0.0373	0.8007	1.0251	0.033*	
C9	0.81677 (19)	0.69444 (19)	0.62641 (14)	0.0316 (4)	
H9	0.8839	0.6937	0.5637	0.038*	
C15	0.1951 (2)	0.91087 (18)	0.45130 (13)	0.0315 (4)	
H15	0.1735	1.0083	0.4141	0.038*	

### Atomic displacement parameters (Å^2^)

**Table:**

	*U*^11^	*U*^22^	*U*^33^	*U*^12^	*U*^13^	*U*^23^
P1	0.01569 (17)	0.01745 (17)	0.01619 (17)	−0.00773 (13)	0.00018 (13)	−0.00564 (13)
P2	0.0302 (2)	0.0301 (2)	0.0236 (2)	−0.01993 (18)	−0.00010 (16)	−0.00870 (16)
F2	0.0362 (5)	0.0439 (6)	0.0369 (5)	−0.0220 (5)	0.0021 (4)	−0.0225 (5)
F6	0.0485 (6)	0.0327 (5)	0.0294 (5)	−0.0224 (5)	−0.0021 (4)	−0.0055 (4)
F4	0.0490 (6)	0.0336 (5)	0.0328 (5)	−0.0257 (5)	−0.0006 (4)	−0.0132 (4)
F5	0.0403 (6)	0.0640 (7)	0.0306 (5)	−0.0381 (5)	0.0065 (4)	−0.0166 (5)
F3	0.0308 (5)	0.0479 (6)	0.0599 (7)	−0.0238 (5)	0.0105 (5)	−0.0308 (5)
O2	0.0238 (5)	0.0203 (5)	0.0248 (5)	−0.0076 (4)	0.0042 (4)	−0.0074 (4)
F1	0.0651 (7)	0.0398 (6)	0.0371 (6)	−0.0275 (5)	−0.0218 (5)	−0.0001 (5)
O1	0.0294 (6)	0.0194 (5)	0.0389 (7)	−0.0107 (5)	0.0116 (5)	−0.0102 (5)
C5	0.0165 (6)	0.0172 (6)	0.0193 (7)	−0.0080 (5)	0.0001 (5)	−0.0053 (5)
C18	0.0214 (7)	0.0237 (7)	0.0199 (7)	−0.0115 (6)	−0.0009 (5)	−0.0058 (6)
C11	0.0157 (6)	0.0226 (7)	0.0173 (6)	−0.0076 (5)	0.0014 (5)	−0.0070 (5)
C17	0.0149 (6)	0.0221 (7)	0.0175 (6)	−0.0070 (5)	−0.0012 (5)	−0.0054 (5)
C4	0.0216 (7)	0.0181 (6)	0.0192 (7)	−0.0060 (5)	−0.0033 (5)	−0.0052 (5)
C1	0.0192 (7)	0.0192 (6)	0.0209 (7)	−0.0089 (5)	0.0005 (5)	−0.0077 (5)
C12	0.0193 (7)	0.0283 (7)	0.0208 (7)	−0.0119 (6)	0.0023 (5)	−0.0087 (6)
C2	0.0182 (7)	0.0185 (7)	0.0260 (7)	−0.0077 (5)	−0.0021 (5)	−0.0054 (6)
C6	0.0210 (7)	0.0230 (7)	0.0219 (7)	−0.0089 (6)	0.0002 (6)	−0.0087 (6)
C19	0.0216 (7)	0.0361 (8)	0.0194 (7)	−0.0169 (6)	0.0012 (6)	−0.0060 (6)
C22	0.0244 (7)	0.0200 (7)	0.0238 (7)	−0.0068 (6)	0.0029 (6)	−0.0053 (6)
C13	0.0201 (7)	0.0415 (9)	0.0212 (7)	−0.0151 (7)	0.0017 (6)	−0.0138 (6)
C3	0.0186 (7)	0.0208 (7)	0.0296 (8)	−0.0061 (6)	0.0011 (6)	−0.0055 (6)
C14	0.0199 (7)	0.0402 (9)	0.0183 (7)	−0.0071 (6)	−0.0011 (6)	−0.0084 (6)
C16	0.0319 (8)	0.0241 (7)	0.0248 (8)	−0.0124 (6)	−0.0042 (6)	−0.0060 (6)
C7	0.0233 (7)	0.0252 (7)	0.0307 (8)	−0.0088 (6)	−0.0061 (6)	−0.0108 (6)
C8	0.0209 (7)	0.0291 (8)	0.0361 (9)	−0.0142 (6)	−0.0014 (6)	−0.0070 (7)
C10	0.0264 (8)	0.0345 (8)	0.0204 (7)	−0.0167 (7)	0.0038 (6)	−0.0107 (6)
C21	0.0264 (8)	0.0255 (8)	0.0293 (8)	−0.0020 (6)	0.0028 (6)	−0.0102 (6)
C20	0.0175 (7)	0.0396 (9)	0.0217 (7)	−0.0071 (6)	0.0029 (6)	−0.0122 (7)
C9	0.0264 (8)	0.0440 (10)	0.0297 (8)	−0.0213 (7)	0.0081 (7)	−0.0108 (7)
C15	0.0356 (9)	0.0268 (8)	0.0251 (8)	−0.0078 (7)	−0.0054 (7)	−0.0018 (6)

### Geometric parameters (Å, º)

**Table:**

P1—C5	1.7867 (14)	C2—H2B	0.9900
P1—C11	1.7910 (14)	C2—C3	1.5227 (19)
P1—C17	1.7930 (14)	C6—H6	0.9500
P1—C1	1.8000 (14)	C6—C7	1.388 (2)
P2—F2	1.6053 (10)	C19—H19	0.9500
P2—F6	1.6054 (10)	C19—C20	1.382 (2)
P2—F4	1.6044 (10)	C22—H22	0.9500
P2—F5	1.6058 (10)	C22—C21	1.384 (2)
P2—F3	1.5990 (10)	C13—H13	0.9500
P2—F1	1.5951 (11)	C13—C14	1.386 (2)
O2—C4	1.2216 (17)	C3—H3A	0.9900
O1—H1	0.8400	C3—H3B	0.9900
O1—C4	1.3140 (17)	C14—H14	0.9500
C5—C6	1.3919 (19)	C14—C15	1.382 (2)
C5—C10	1.3977 (19)	C16—H16	0.9500
C18—H18	0.9500	C16—C15	1.388 (2)
C18—C17	1.3964 (19)	C7—H7	0.9500
C18—C19	1.391 (2)	C7—C8	1.384 (2)
C11—C12	1.396 (2)	C8—H8	0.9500
C11—C16	1.397 (2)	C8—C9	1.384 (2)
C17—C22	1.3975 (19)	C10—H10	0.9500
C4—C3	1.496 (2)	C10—C9	1.385 (2)
C1—H1A	0.9900	C21—H21	0.9500
C1—H1B	0.9900	C21—C20	1.390 (2)
C1—C2	1.5352 (19)	C20—H20	0.9500
C12—H12	0.9500	C9—H9	0.9500
C12—C13	1.386 (2)	C15—H15	0.9500
C2—H2A	0.9900		
			
C5—P1—C11	110.19 (6)	C3—C2—C1	110.82 (12)
C5—P1—C17	110.68 (6)	C3—C2—H2A	109.5
C5—P1—C1	107.80 (6)	C3—C2—H2B	109.5
C11—P1—C17	107.83 (6)	C5—C6—H6	120.3
C11—P1—C1	109.51 (6)	C7—C6—C5	119.50 (13)
C17—P1—C1	110.83 (7)	C7—C6—H6	120.3
F2—P2—F6	89.92 (5)	C18—C19—H19	119.7
F2—P2—F5	89.94 (5)	C20—C19—C18	120.63 (14)
F6—P2—F5	89.47 (6)	C20—C19—H19	119.7
F4—P2—F2	179.53 (6)	C17—C22—H22	120.3
F4—P2—F6	89.72 (5)	C21—C22—C17	119.49 (14)
F4—P2—F5	89.76 (5)	C21—C22—H22	120.3
F3—P2—F2	89.89 (5)	C12—C13—H13	120.0
F3—P2—F6	89.71 (6)	C12—C13—C14	119.99 (14)
F3—P2—F4	90.41 (5)	C14—C13—H13	120.0
F3—P2—F5	179.17 (7)	C4—C3—C2	115.15 (12)
F1—P2—F2	89.99 (6)	C4—C3—H3A	108.5
F1—P2—F6	179.28 (6)	C4—C3—H3B	108.5
F1—P2—F4	90.37 (6)	C2—C3—H3A	108.5
F1—P2—F5	89.82 (6)	C2—C3—H3B	108.5
F1—P2—F3	91.00 (6)	H3A—C3—H3B	107.5
C4—O1—H1	109.5	C13—C14—H14	119.7
C6—C5—P1	120.87 (11)	C15—C14—C13	120.53 (14)
C6—C5—C10	120.30 (13)	C15—C14—H14	119.7
C10—C5—P1	118.10 (11)	C11—C16—H16	120.4
C17—C18—H18	120.4	C15—C16—C11	119.24 (15)
C19—C18—H18	120.4	C15—C16—H16	120.4
C19—C18—C17	119.17 (13)	C6—C7—H7	119.9
C12—C11—P1	119.73 (11)	C8—C7—C6	120.18 (14)
C12—C11—C16	120.37 (13)	C8—C7—H7	119.9
C16—C11—P1	119.58 (11)	C7—C8—H8	119.8
C18—C17—P1	121.95 (11)	C7—C8—C9	120.35 (14)
C18—C17—C22	120.34 (13)	C9—C8—H8	119.8
C22—C17—P1	117.45 (11)	C5—C10—H10	120.3
O2—C4—O1	124.32 (13)	C9—C10—C5	119.44 (14)
O2—C4—C3	123.95 (13)	C9—C10—H10	120.3
O1—C4—C3	111.72 (12)	C22—C21—H21	119.8
P1—C1—H1A	109.1	C22—C21—C20	120.45 (14)
P1—C1—H1B	109.1	C20—C21—H21	119.8
H1A—C1—H1B	107.8	C19—C20—C21	119.90 (14)
C2—C1—P1	112.67 (9)	C19—C20—H20	120.1
C2—C1—H1A	109.1	C21—C20—H20	120.1
C2—C1—H1B	109.1	C8—C9—C10	120.20 (14)
C11—C12—H12	120.2	C8—C9—H9	119.9
C13—C12—C11	119.56 (14)	C10—C9—H9	119.9
C13—C12—H12	120.2	C14—C15—C16	120.28 (15)
C1—C2—H2A	109.5	C14—C15—H15	119.9
C1—C2—H2B	109.5	C16—C15—H15	119.9
H2A—C2—H2B	108.1		
			
P1—C5—C6—C7	168.72 (11)	C17—P1—C11—C12	−92.86 (12)
P1—C5—C10—C9	−168.71 (12)	C17—P1—C11—C16	80.66 (13)
P1—C11—C12—C13	172.55 (11)	C17—P1—C1—C2	−77.68 (11)
P1—C11—C16—C15	−172.12 (12)	C17—C18—C19—C20	0.8 (2)
P1—C17—C22—C21	173.20 (12)	C17—C22—C21—C20	0.3 (2)
P1—C1—C2—C3	−179.11 (10)	C1—P1—C5—C6	−96.79 (12)
O2—C4—C3—C2	−9.1 (2)	C1—P1—C5—C6	−96.79 (12)
O1—C4—C3—C2	171.88 (13)	C1—P1—C5—C10	73.39 (13)
C5—P1—C11—C12	146.24 (11)	C1—P1—C11—C12	27.83 (13)
C5—P1—C11—C16	−40.23 (13)	C1—P1—C11—C12	27.83 (13)
C5—P1—C17—C18	−131.04 (12)	C1—P1—C11—C16	−158.65 (11)
C5—P1—C17—C22	54.76 (13)	C1—P1—C17—C18	−11.48 (14)
C5—P1—C1—C2	43.58 (12)	C1—P1—C17—C18	−11.48 (14)
C5—C6—C7—C8	−0.4 (2)	C1—P1—C17—C22	174.32 (11)
C5—C10—C9—C8	−0.2 (2)	C1—C2—C3—C4	−77.18 (16)
C18—C17—C22—C21	−1.1 (2)	C12—C11—C16—C15	1.4 (2)
C18—C19—C20—C21	−1.6 (2)	C12—C13—C14—C15	1.8 (2)
C11—P1—C5—C6	143.74 (11)	C6—C5—C10—C9	1.5 (2)
C11—P1—C5—C10	−46.07 (13)	C6—C7—C8—C9	1.7 (2)
C11—P1—C17—C18	108.38 (12)	C19—C18—C17—P1	−173.47 (11)
C11—P1—C17—C22	−65.82 (13)	C19—C18—C17—C22	0.6 (2)
C11—P1—C1—C2	163.47 (10)	C22—C21—C20—C19	1.1 (2)
C11—C12—C13—C14	−0.7 (2)	C13—C14—C15—C16	−1.4 (2)
C11—C16—C15—C14	−0.2 (2)	C16—C11—C12—C13	−0.9 (2)
C17—P1—C5—C6	24.57 (14)	C7—C8—C9—C10	−1.5 (3)
C17—P1—C5—C10	−165.24 (11)	C10—C5—C6—C7	−1.2 (2)

### Hydrogen-bond geometry (Å, º)

**Table:**

*D*—H···*A*	*D*—H	H···*A*	*D*···*A*	*D*—H···*A*
O1—H1···O2^i^	0.84	1.80	2.6285 (15)	171
C1—H1*A*···F2	0.99	2.48	3.455 (2)	168
C1—H1*A*···F3	0.99	2.50	3.1656 (19)	124
C22—H22···F4^ii^	0.95	2.51	3.3924 (18)	155

Symmetry codes: (i) −*x*+1, −*y*, −*z*+2; (ii) *x*, *y*+1, *z*.
