# Supplementary figures and images for: Crystal structure of (3-carb­oxy­prop­yl)tri­phenyl­phospho­nium hexa­fluorido­phosphate
Source: Acta Crystallogr Sect E Struct Rep Online. 2014 Oct 24;70(Pt 11):o1197–8. doi: 10.1107/S160053681402323X (PMC4257321; doi:10.1107/S160053681402323X)

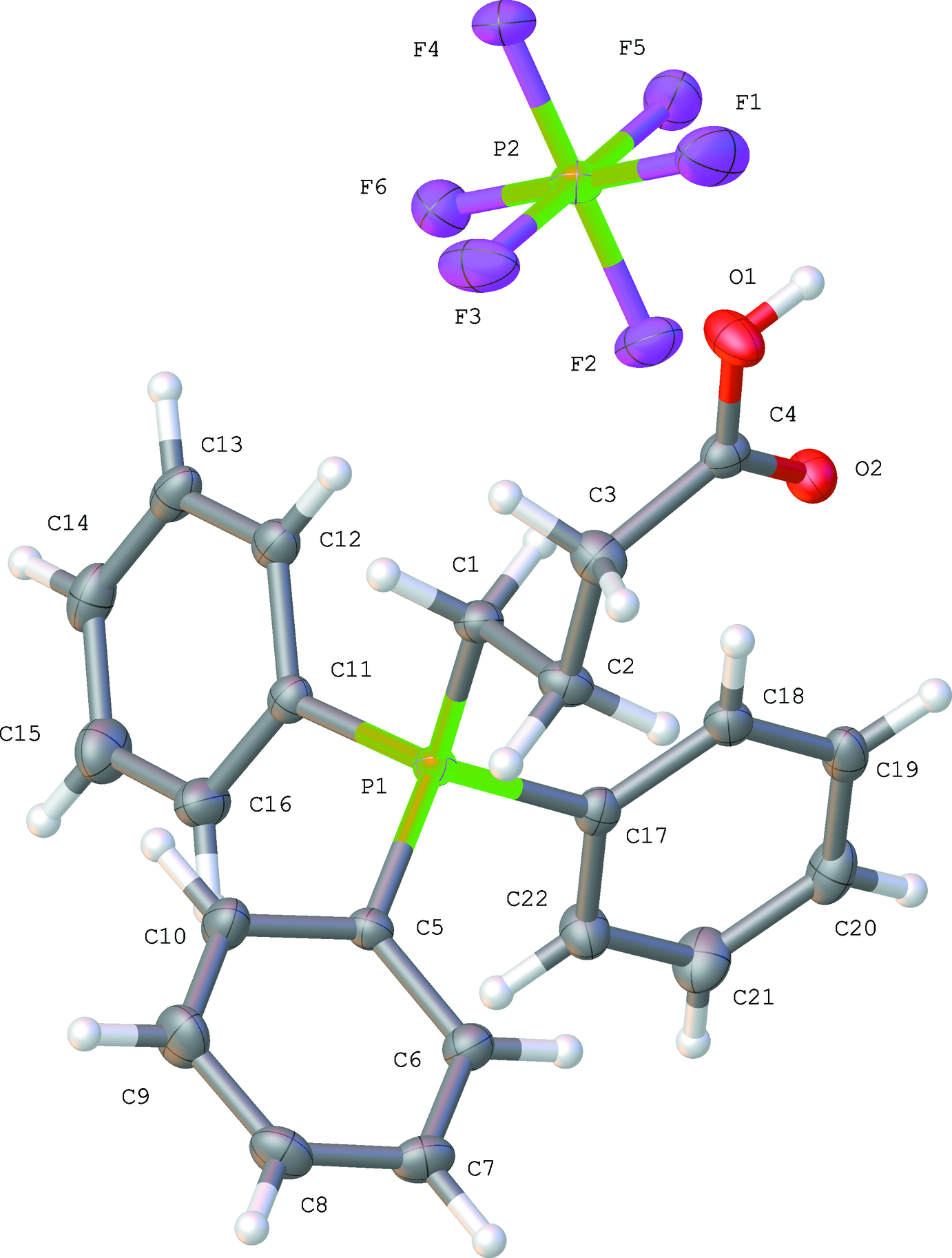

Supplement: Supplementary file 4 [file e-70-o1197-fig1.tif]
